# Maternal Obesity and Neonatal Metabolic Health: Insights Into Insulin Resistance

**DOI:** 10.7759/cureus.55923

**Published:** 2024-03-10

**Authors:** Amit D Sonagra, Deepak Parchwani, Ragini Singh, Sagar Dholariya, Anita Motiani, DVSS Ramavataram

**Affiliations:** 1 Biochemistry, All India Institute of Medical Sciences, Rajkot, IND; 2 Biochemistry, Smt. B. K. Shah Medical Institute & Research Centre, Vadodara, IND

**Keywords:** quicki, homa-ir, cord blood glucose, insulin sensitivity, pregnancy, maternal obesity, insulin resistance, cord blood insulin

## Abstract

Background

Maternal obesity is a global health concern that leads to metabolic alterations in the offspring, making them vulnerable to metabolic disorders in adulthood. Early identification of such neonates would provide opportunities to positively alter modifiable risk factors for non-communicable diseases (NCDs) to prevent their occurrence later in life.

Objectives

This study aimed to assess and contrast insulin resistance (IR) levels in neonates born to mothers with obesity and those born to healthy, non-obese mothers.

Methods

This case-control study was conducted after approval from the institutional ethics committee. A total of 98 healthy, non-obese pregnant females were included in Group 1, and 68 obese pregnant females were included in Group 2. The participants were followed up until delivery and cord blood samples were collected after delivery. Neonatal glucose and insulin concentrations were estimated, and indices of IR such as homeostatic model assessment for insulin resistance (HOMA-IR), quantitative insulin-sensitivity check index (QUICKI), and glucose-to-insulin ratio were calculated. Neonatal IR indices and anthropometric measurements were compared between the groups using the Z test and correlated with the maternal pre-pregnancy body mass index (BMI) using Pearson’s correlation. Additionally, Pearson’s correlations were examined between neonatal IR indices and anthropometric measurements. Statistical significance was set at p <0.05.

Results

Neonates in Group 2 exhibited significantly higher anthropometric parameters and IR indices than those in Group 1. A statistically significant positive correlation was identified between maternal pre-pregnancy BMI, neonatal anthropometric parameters, and IR. Furthermore, a statistically significant positive correlation was observed between neonatal IR and the anthropometric parameters.

Conclusion

Neonates born to obese mothers exhibited higher anthropometric parameters and insulin resistance than those born to non-obese, healthy mothers. Assessment of IR at birth can help identify neonates who are at higher risk of developing NCD in later life. Timely promotion of a healthy lifestyle can reduce the occurrence of NCDs in later life.

## Introduction

Globally, the prevalence of overweight and obesity has sharply increased, affecting over 1.9 billion adults, with 650 million classified as obese [1]. This trend spans age groups, genders, and socioeconomic backgrounds in both developed and developing nations [2]. In India, traditionally grappling with malnutrition, there has been a notable shift, as both rural and urban areas have witnessed an increase in obesity. A Lancet study revealed that India has the third-highest obese and overweight population globally, following the United States and China. Contributing factors include changing dietary patterns, urbanization, sedentary lifestyles, and genetic predispositions [3]. The National Family Health Survey-5 in India (2019-2021) reported a 24% prevalence of overweight and obesity in women and 22.9% in men aged 15-49 years, with higher rates in urban areas. In addition, India exhibited a significant proportion of women with a higher waist-to-hip ratio. The country has the highest incidence of overweight and obese pregnant women globally, totaling 4.3 million individuals, comprising 11.1% of the global demographic [3,4].

Obesity involves persistent inflammation initiated by the excessive accumulation of nutrients in adipocytes, causing intracellular stress and inflammatory cascades. Adipocytes release various substances, including hormones and cytokines, leading to dysregulation of pro-inflammatory and anti-inflammatory elements. This disruption affects the insulin signaling pathway, resulting in diminished insulin sensitivity and the onset of insulin resistance (IR) [5]. Pregnancy inherently entails a condition characterized by IR, and obesity further adds to this. Increased IR in obese mothers can modify the fetal metabolic milieu, thereby influencing fetal metabolic programming [6]. This modification prompts fetal epigenetic alterations that affect gene expression patterns. These stable epigenetic modifications transmitted during cellular proliferation have the potential to induce changes in the physiological phenotype without concurrent alterations in the nucleotide sequence [7].

Deviations in fetal metabolic programming may manifest as variations in serum insulin levels and modifications in insulin sensitivity patterns. Such alterations in these parameters increase the likelihood of developing metabolic syndrome, with potential consequences including obesity, cardiovascular diseases, and diabetes mellitus during adulthood [7,8]. A substantial proportion of risk factors associated with metabolic syndrome are amenable to modification, including dietary choices, physical activity, lifestyle, tobacco use, alcohol consumption, and exposure to mental, physical, or social stress [9]. We hypothesized that newborns of obese mothers may exhibit heightened IR compared to those of healthy, lean mothers. Identifying susceptible individuals at birth would enable timely intervention to positively alter modifiable risk factors for non-communicable diseases (NCDs), thereby mitigating or delaying the onset of IR and its associated consequences in adulthood.

In the present study, an attempt was made to identify alterations in insulin sensitivity among newborns of obese mothers compared to newborns of healthy, lean mothers.

## Materials and methods

This case-control study was carried out in the obstetrics and gynecology, pediatrics, and clinical biochemistry laboratory of Gujarat Medical Education and Research Society Medical College and Hospital, Patan, India, from January 2018 to December 2021. Institutional ethics committee approval was obtained before the initiation of the study. Pregnant women admitted to the obstetrics and gynecology department for imminent labor were recruited in the study by non-probability consecutive sampling after obtaining written informed consent. Maternal vital parameters were also recorded. Maternal pre-pregnancy weight was obtained by history, and height was noted from the case record of the first antenatal visit. Mothers with a pre-pregnancy body mass index (BMI) >30 kg/m^2^ were considered obese [10]. A total of 68 obese mothers (cases) and 98 age-matched healthy non-obese mothers (controls) without any illness were included in the study. Controls were assigned to Group 1, and cases were assigned to Group 2. Women with a history of hypertension, diabetes mellitus, insulin therapy, hypoglycemic or hypolipidemic drug intake, smoking, alcoholism, liver, cardiac, or renal diseases, or any other major illness were excluded from the study. Women with twins or multiple gestations were excluded from this study.

The participants were followed up until delivery. Five milliliters of umbilical venous blood were drawn in an ethylenediamine tetraacetic acid (EDTA) vacutainer from a double-clamped cord immediately after delivery of the placenta under appropriate aseptic precautions. Plasma was separated by centrifugation at 1,200 g for 10 minutes. Glucose was analyzed soon after plasma separation using the glucose oxidase-peroxidase method (Erba Chem 5x analyzer, Erba-Mannheim, Mannheim, Germany) with intra-assay and inter-assay coefficients of variation (CV) of 2.5% and 2.34%, respectively. The rest of the separated plasma was collected in a properly labeled aliquot and stored at -20°C or analyzed immediately for insulin. However, all samples were analyzed within three days of collection.

The samples were thawed to room temperature, and the insulin concentration was analyzed in batches. Plasma insulin was estimated using an enzyme-linked immunosorbent assay (ELISA) kit (Ray Biotech Inc., Peachtree Corners, GA and Erba LisaScan ELISA reader, Erba-Mannheim) with intra-assay and inter-assay coefficients of variation (CV) of 7.21% and 9.45%, respectively. The IR indices, which include homeostasis model assessment (HOMA-IR), quantitative insulin sensitivity check index (QUICKI), glucose-to-insulin ratio (GIR), and log insulin, were calculated from the values of plasma glucose (PG) and plasma insulin (PI) concentrations based on the formulas: HOMA IR = (PG in mg/dl x PI in μIU/ml) / 405; QUICKI = 1/ (log PG in mg/dl + log PI in μIU/ml); GIR = (PG in mg/dl) / (PI in μIU/ml) [11].

The anthropometric parameters of neonates were assessed within 12 hours of birth. Weight measurements were performed using a calibrated weighing scale. Length measurements were conducted using an infantometer with the infant in the supine position, ensuring proper alignment of the body to the board, extension of the knees, and parallel positioning of the feet to the footboard. Head circumference (HC), mid-upper arm circumference (MUAC), and chest circumference (CC) were measured using a non-stretchable measuring tape. The HC was determined by encircling the measuring tape above the superciliary arch on the anterior aspect, laterally above the auricles, and posteriorly at the level of the occipital protuberance. The MUAC was measured at the midpoint between the acromion process of the scapula and the olecranon process of the ulna. The CC was measured at the nipple line during mid-expiration, rounded to the nearest 0.1 cm [12].

Data compilation and statistical analysis

All data were collected and compiled using Microsoft Excel 2019 (Microsoft Corp., Redmond, WA). Statistical analysis was performed using IBM SPSS Statistics for Windows, version 26.0 (IBM Corp., Armonk, NY), using appropriate statistical tests. The mean and standard deviation were calculated for continuous variables. Frequencies and percentages were calculated for qualitative parameters. The Z-test was used for comparison of continuous variables, and the chi-square test was used to analyze qualitative parameters. Pearson’s correlation was used to assess the correlation between continuous parameters. Statistical significance was set at p <0.05.

## Results

No significant difference was observed in maternal age or gestational age between the study groups (Table 1). There was a significantly higher requirement for a lower segment cesarean section (LSCS) for the birth of the baby in obese mothers than in lean mothers (Table 2). Significantly higher anthropometric parameters and IR indices were observed in the neonates of Group 2 than in those of Group 1 (Table 3). Table 4 shows a highly significant correlation of maternal pre-pregnancy BMI with neonatal anthropometric parameters, and IR indices. As shown in Table 5, there was a significant positive correlation between neonatal IR and neonatal anthropometric parameters. 

**Table 1:** Demographic and anthropometric parameters of non-obese and obese mothers

Parameter	Unit	Non-obese mothers	Obese mothers	p-value for Z-test
		(Mean ± SD)	(Mean ± SD)	
Maternal age	Years	25.04 ± 3.85	26.35 ± 4.58	0.06
Pre-pregnancy BMI	kg/m^2^	21.86 ± 2.31	33.76 ± 2.45	< 0.001
Gestational age	Weeks	37.17 ± 1.11	37.32 ± 0.8	0.31

**Table 2:** Comparison between the mode of delivery and gender among the study participants

Parameter	Unit	Non-obese mothers	Obese mothers	p-value for chi-square test
		Frequency (%)	Frequency (%)	
Mode of delivery	Normal labor	66 (67.34%)	35 (51.47%)	0.039
	Lower segment cesarean section (LSCS)	32 (32.66%)	33 (48.53%)	
Gender of the offspring	Male	51 (52.04%)	35 (51.47%)	0.942
	Female	47 (47.96%)	33 (48.53%)	

**Table 3:** Comparison of anthropometric and metabolic parameters of neonates born to non-obese (Group 1: n=98) and obese (Group 2: n=68) mothers

Parameter	Group 1	Group 2	p-value for Z-test
	(Mean ± SD)	(Mean ± SD)	
Birth weight (kg)	2.64 ± 0.77	2.96 ± 0.86	0.00000001
Birth length (cm)	45.32 ± 3.07	47.74 ± 3.5	0.000011
Head circumference (cm)	33.10 ± 0.68	33.63 ± 0.96	0.00015
Chest circumference (cm)	30.22 ± 1.49	31.25 ± 1.58	0.00001
Mid-upper arm circumference (cm)	8.84 ± 1.07	10.11 ± 1.43	0.00000001
Umbilical cord insulin (μIU/ml)	6.57 ± 1.86	8.09 ± 2.36	0.000018
Umbilical cord glucose (mg/dL)	80.15 ± 11.14	77.45 ± 7.04	0.06
Homeostasis model assessment (HOMA-IR)	1.28 ± 0.31	1.55 ± 0.48	0.000087
Quantitative insulin sensitivity check index (QUICKI)	0.371 ± 0.016	0.361 ± 0.018	0.0003
Glucose-to-insulin ratio (GIR)	13.48 ± 5.05	10.48 ± 3.45	0.00001
Log insulin	0.80 ± 0.13	0.89 ± 0.13	0.000024

**Table 4:** Pearson’s correlation of maternal pre-pregnancy BMI with neonatal anthropometric and biochemical parameters

Parameter (n=166)	r value	p-value
Birth weight	0.443	0.00000001
Birth length	0.456	0.0000102
Head circumference	0.335	0.000001
Chest circumference	0.340	0.000001
Mid-upper arm circumference	0.382	0.000000001
Umbilical cord insulin	0.327	0.0000167
Umbilical cord glucose	-0.120	0.122
Homeostasis model assessment (HOMA-IR)	0.319	0.000024
Quantitative insulin sensitivity check index (QUICKI)	-0.282	0.000381
Glucose to Insulin ratio (GIR)	-0.310	0.0026

**Table 5:** Pearson’s correlation between neonatal insulin resistance (homeostasis model assessment (HOMA-IR)) and neonatal anthropometric parameters

Parameter (n=166)	r value	p-value
Birth weight	0.334	0.000011
Birth length	0.280	0.000253
Head circumference	0.328	0.000016
Chest circumference	0.308	0.000053
Mid-upper arm circumference	0.321	0.000024

## Discussion

A global epidemic of obesity is prevalent in both developing and developed nations. A significant portion of the overall health sector budget is dedicated to addressing complications arising from obesity. Cardiovascular diseases, hypertension, diabetes mellitus, and their complications are frontrunners among obesity-related morbidities that necessitate substantial allocation of resources, manpower, and man hours [1,2,13]. Numerous studies have established a correlation between obesity and insulin resistance as well as inflammation. The coexistence of these conditions during pregnancy adds a layer of complexity to the scenario, with documented associations with heightened maternal and fetal morbidities and mortality. Additionally, it is noted to influence various facets of fetal growth and metabolism [6,13,14]. This study aimed to investigate alterations in insulin sensitivity among newborns born to mothers with obesity.

Studies have highlighted age as a notable factor influencing insulin sensitivity, indicating a progressive rise in IR with increasing age [3,15]. Pregnancy inherently induces a state of IR, with a concurrent escalation in IR as gestation progresses [14]. Investigations have reported a notable gender-based disparity in insulin sensitivity among neonates, with female offspring demonstrating higher IR at birth compared to their male counterparts [16]. In the present study, there was no significant difference in the age of the mother, gestational age, or gender of the offspring among study groups.

We identified a substantial increase in birth weight, along with other neonatal anthropometric parameters, in neonates delivered by obese mothers. Additionally, a significant elevation in umbilical cord insulin levels was noted in these cases. The established association between IR and obesity, corroborated by numerous studies, elucidates that heightened IR in obese mothers contributes to augmented hepatic glucose production and lipolysis [10,17-19]. Consequently, there is an elevated flow of nutrients to the fetus, leading to an increase in fetal insulin production [14]. Given insulin's anabolic nature, such neonates exhibit heightened fetal growth and an augmented fat mass [19,20]. It is noteworthy that neonates with increased anthropometric measurements are predisposed to cephalo-pelvic disproportion, potentially resulting in obstructed labor. Consequently, operative interventions, particularly in the form of a cesarean section (LSCS), are more frequently required for the delivery of these infants [21].

Increased IR in mothers with obesity induces alterations in the metabolic milieu of the intrauterine fetus. These changes have the potential to induce epigenetic modifications in the fetus, thereby influencing its metabolic programming [8,22]. Key mechanisms involved in this process include DNA methylation, histone modification, and chromatin remodeling. These mechanisms regulate gene expression at levels of pre-transcription, transcription, and translation. Noteworthy alterations in gene expression are discernible in genes related to adipose tissue development, hyperphagia, regulation of energy balance, lipid metabolism, insulin-associated signaling, and the development of adipocytokines and proinflammatory factors such as leptin and adiponectin, as well as pancreatic islet beta cell development. Collectively, these changes contribute to diminished insulin sensitivity [8,23,24]. The study further identified elevated IR in the cases, as indicated by elevated HOMA-IR and reduced QUICKI and GIR, which are aligned with previous studies [25,26]. 

We observed a significant positive correlation of maternal BMI with neonatal anthropometric parameters such as birth weight and length, along with head, chest, and mid-upper arm circumferences. Our observations revealed a concurrent increase in IR with elevated maternal BMI, exemplified by the positive correlation of maternal BMI with umbilical cord blood insulin levels and HOMA-IR, and a negative correlation with QUICKI and GIR. This signifies an association between maternal obesity and fetal hyperinsulinemia, accompanied by IR. Furthermore, a significant positive correlation was identified between the IR index (HOMA-IR) and neonatal anthropometric parameters, highlighting the anabolic influence of insulin on higher birth weight and other anthropometric measures. These findings align with those of prior studies conducted by Catalano et al. [27], Ikedionwu et al. [26], and Lewandowska [28]. These studies showed a significantly higher occurrence of macrosomic babies among obese mothers [26,28] and significantly higher HOMA-IR in babies of obese mothers compared with healthy lean mothers [27]. They also found a significant association between fetal adiposity and IR [27].

The present study endeavors to identify neonates exhibiting elevated IR right from birth, a critical juncture for potential intervention. Such early detection holds immense promise for averting the onset of complications related to IR in adulthood, encompassing metabolic syndrome, obesity, cardiovascular diseases, and diabetes mellitus [8]. Through comprehensive counseling for both parents and children and the adoption of tailored modifications in diet, lifestyle, and physical activity, the study underscores the potential to bolster insulin sensitivity. By embracing these interventions early on, there is a tangible prospect of preventing or significantly delaying the emergence of complications linked to IR. In essence, the insights gleaned from this study hold the key to not only addressing IR in neonates but also to effectively mitigating modifiable risk factors associated with NCDs through informed counseling and proactive lifestyle changes [9,29].

Limitations of the study

The study was conducted at a single center and involved a limited number of participants. The investigation lacks a follow-up assessment of the cases in their adult lives to evaluate the status of IR. A prospective study involving a larger cohort of subjects from multiple centers would be warranted to comprehensively analyze the impact of maternal obesity on the health and metabolic profile of the offspring.

## Conclusions

The present study assessed the degree of IR in neonates born to obese mothers compared to those born to non-obese, healthy mothers. Our findings indicate elevated anthropometric parameters and IR in neonates born to obese mothers compared with those born under normal pregnancy conditions. Estimation of IR at the time of birth enables medical practitioners to identify neonates who are at a higher risk of developing metabolic syndrome and associated complications in adulthood. The implementation of counseling interventions designed to promote a healthy lifestyle may emerge as a crucial preventive strategy against the initiation of lifestyle-related diseases and subsequent complications. This proactive approach could become a firm step in reducing the burden of metabolic diseases worldwide.
